# Construction of competing endogenous RNA networks from paired RNA-seq data sets by pointwise mutual information

**DOI:** 10.1186/s12864-019-6321-x

**Published:** 2019-12-24

**Authors:** Chaowang Lan, Hui Peng, Gyorgy Hutvagner, Jinyan Li

**Affiliations:** 1 Advanced Analytics Institute, Faculty of Engineering and IT, University of Technology Sydney, PO Box 123, Broadway, NSW, 2007 Australia; 2 School of Biomedical Engineering, Faculty of Engineering and IT, University of Technology Sydney, PO Box 123, Broadway, NSW, 2007 Australia

**Keywords:** Competing endogenous RNA, Pointwise mutual information, Competition rule

## Abstract

**Background:**

A long noncoding RNA (lncRNA) can act as a competing endogenous RNA (ceRNA) to compete with an mRNA for binding to the same miRNA. Such an interplay between the lncRNA, miRNA, and mRNA is called a ceRNA crosstalk. As an miRNA may have multiple lncRNA targets and multiple mRNA targets, connecting all the ceRNA crosstalks mediated by the same miRNA forms a ceRNA network. Methods have been developed to construct ceRNA networks in the literature. However, these methods have limits because they have not explored the expression characteristics of total RNAs.

**Results:**

We proposed a novel method for constructing ceRNA networks and applied it to a paired RNA-seq data set. The first step of the method takes a competition regulation mechanism to derive candidate ceRNA crosstalks. Second, the method combines a competition rule and pointwise mutual information to compute a competition score for each candidate ceRNA crosstalk. Then, ceRNA crosstalks which have significant competition scores are selected to construct the ceRNA network. The key idea, pointwise mutual information, is ideally suitable for measuring the complex point-to-point relationships embedded in the ceRNA networks.

**Conclusion:**

Computational experiments and results demonstrate that the ceRNA networks can capture important regulatory mechanism of breast cancer, and have also revealed new insights into the treatment of breast cancer. The proposed method can be directly applied to other RNA-seq data sets for deeper disease understanding.

## Background

Long non-coding RNAs (lncRNAs) are involved in a variety of biological functions [1]. However, not much is known about the functions and regulatory mechanisms of non-coding RNAs with other types of RNAs [2]. Some early studies [3, 4] found that a RNA can influence the expression level of other RNAs by competing to bind to the same miRNA. Based on these early findings, Pandolfi proposed a competing endogenous RNA (ceRNA) hypothesis [5]. This ceRNA hypothesis stated that non-coding RNAs and coding RNAs would widely compete with mRNAs for binding to the same miRNAs. This ceRNA hypothesis not only provides a reasonable justification for the presence of lncRNA, it also provides a new and global function map of lncRNA [6], explaining the regulatory function of 3^′^ UTRs [5]. Recent experiments have provided new evidence for this hypothesis. For example, *BRAFP1* can compete with gene *BRAF* for binding to the same miRNA hsa-miR-543 in lymphoma [7]; *PTENP1* can compete with gene *PTEN* for binding to the same miRNA hsa-miR-17-5p in hepatocellular carcinoma [8]. Both non-coding RNAs and coding RNAs can act as ceRNAs according to the ceRNA hypothesis. We focus on the investigation of long non-coding ceRNAs in this work.

When a lncRNA acts as a ceRNA to compete with an mRNA for binding to the same miRNA, this interplay between the lncRNA, miRNA, and mRNA is called a ceRNA crosstalk. An miRNA may have multiple target lncRNAs and it can also regulate several different mRNAs, therefore, there can exist many crosstalks mediated by this miRNA to form a ceRNA network. Such a network is useful for detecting cancer biomarkers [9], patterns for early diagnosis [10], and new concepts for cancer treatment [11].

Every lncRNA in a ceRNA network has three common characteristics [5]. First, changes in the ceRNA expression levels are wide, or they are highly differentially expressed, between tumor and normal samples. Second, the lncRNA is the primary target of the miRNA. Third, the relationships between the lncRNA, miRNA, and mRNA should obey a competition rule in the ceRNA network. The competition rule states that when the expression level of the ceRNA is very high, the ceRNA can compete for binding to the miRNA and decrease the expression level of the miRNA. Since miRNA has a low expression level, less number of miRNAs bind to its target mRNA. Therefore, the expression level of the mRNA becomes high. In contrast, when the expression level of the ceRNA is very low, the expression level of the miRNA will be high; a high expression level of miRNA leads to a low expression level of mRNA.

Many methods for constructing ceRNA networks have been developed and they can be grouped into two categories. As the ceRNA is the primary target of miRNA, the first category of method is based on predicting the target of the miRNA. Traditional methods apply the sequence alignment and the free energy models to discover the primary targets of miRNAs, such as the method TargetScan [12]. However, these methods have a high false positive rate. Later methods employ extra data sets and multiple algorithms to decrease the false positive rate, for example, Sardina’s method [13]. These methods only apply the sequence of miRNA and miRNA targets and do not calculate the expression relationship between miRNAs and miRNA targets. Thus, these methods still have a high false positive rate. Xia’s method identifies the overexpressed lncRNAs from the expression data, but do not consider the competitive relationship between the lncRNA, miRNA, and mRNA [14]. Several methods utilize the Pearson coefficient to find out the competitive relationship between lncRNA, miRNA, and mRNA, e.g., Paci’s method [15]. However, the Pearson coefficient is not suitable for measuring non-linear relationship. An miRNA could bind to multiple targets, the competitive relationship between RNAs is not always linear. These methods neglect the ceRNA networks which pose non-linear relationships. A few methods can measure the non-linear relationship between lncRNA, miRNA, and mRNA but do not consider the overexpressed RNAs, for example, Zhou’s method [16] and Zhang’s method [17]. These methods could identify a lot of ceRNA networks but a few ceRNA networks regulating cancer processes. Other methods such as Chiu’s method [18] discover the pair-wised relationship between two RNAs then use the pair-wised relationship to construct the ceRNA network. The pair-wised relationship is the relationship between two RNAs rather than the competitive relationship between lncRNA, miRNA, and mRNA. The ceRNA network reflects the competition relationship between lncRNA, miRNA, and mRNA. Using these methods to construct ceRNA network may produce some false positives of ceRNA networks. Above all, these two types of methods for predicting ceRNA networks have their limitations. A novel method is demanded to improve the predictions.

We propose a novel method for constructing ceRNA networks from paired RNA-seq data sets. This method identifies the over expressed lncRNAs from the lncRNA expression data of the normal and tumor samples. Thus, we can identify the ceRNA network related to breast cancer. Then, the competitive relationships between the lncRNAs, miRNAs, and mRNAs are established by using the expression levels of the lncRNAs, miRNAs, and mRNAs in the tumor samples. We combine the competition rule and pointwise mutual information to calculate a competition score for each of the ceRNA crosstalks. As an miRNA can have many ceRNAs and can bind to multiple mRNAs, the competitive relationship between lncRNA, miRNA, and mRNA is non-linear. Pointwise mutual information is suitable for measuring the complex point-to-point competitive relationship between RNAs.

## Results

We report two important ceRNA networks related to breast cancer and reveal their characteristics. We also report how these ceRNA networks play vital roles in KEGG pathways. Comparison results with the literature construction methods are presented at the Additional file 1.

### Two important ceRNA networks related to breast cancer

Our method identified 352 mRNAs, 24 miRNAs, and 136 lncRNAs which are differentially expressed between the tumor and normal tissues. As there are 4 of these miRNAs which do not have any predicted target RNAs in the RNAwalker2.0 database, ceRNA networks mediated by the remaining 20 miRNAs which have target RNAs in the database are constructed. The 20 miRNAs are: hsa-miR-200a-5p, hsa-miR-203a-3p, hsa-miR-33a-5p, hsa-miR-21-3p, hsa-miR-183-5p, hsa-miR-144-5p, hsa-miR-145-5p, hsa-miR-184, hsa-miR-451a, hsa-miR-9-3-5p, hsa-miR-182-5p, hsa-miR-940, hsa-miR-375, hsa-miR-5683, hsa-miR-3677-3p, hsa-miR-429, hsa-miR-486-2-5p, hsa-miR-210-3p, hsa-miR-335-5p, hsa-miR-196a-2-5p, hsa-miR-21-5p, hsa-miR-378a-3p, hsa-miR-3065-5p, and hsa-miR-142-3p. The total number of candidate ceRNA crosstalks mediated by these 20 miRNAs is 75501.

**Fig. 1:**
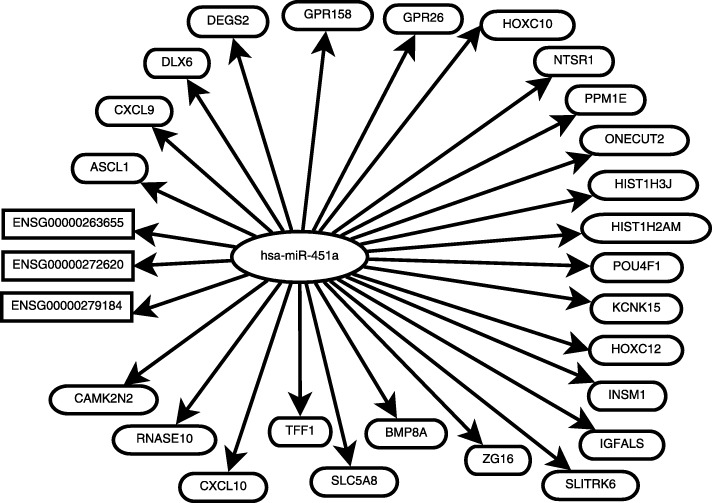
A ceRNA network mediated by hsa-miR-451a. The rectangle and oval boxes contain the names of lncRNAs and mRNAs, respectively

To narrow down the study, we focus our analysis on two significant ceRNA networks: one is mediated by hsa-miR-451a, and the other is mediated by hsa-miR-375. These two miRNAs have a vital role in regulating breast cancer as reported in literature [19, 20], but their ceRNA networks have not been investigated previously. Our pointwise mutual information based method detected 132 candidate ceRNA crosstalks mediated by hsa-miR-451a and 1547 candidate ceRNA crosstalks mediated by hsa-miR-375. Of them, 25 candidate ceRNA crosstalks mediated by hsa-miR-451a have significant competition scores and only 273 candidate ceRNA crosstalks mediated by hsa-miR-375. We use these ceRNA crosstalks which have significant competition scores to construct the ceRNA networks. Fig. 1 is the ceRNA network mediated by hsa-miR-451a and Fig. *S*2 (in the Additional file 1) presents the ceRNA network mediated by hsa-miR-375.

### Characteristics of the two ceRNA networks

The two ceRNA networks are satisfied with the three characteristics of ceRNA networks: (1) the expression level of every lncRNA between the normal and tumor samples is highly differential, (2) every lncRNA is a target of the miRNA, and (3) the expression levels of lncRNA, mRNA and miRNA follow the competition rule. The absolute fold change of these lncRNAs in ceRNA crosstalks mediated by hsa-miR-451a and hsa-miR-375 are larger than 3.0 and the *p*-values are smaller than 0.01. This means that these lncRNAs are over-expressed and satisfy the first point of characteristics of a ceRNA network. Table *S*3 presents the detailed expression fold change and the *p*-values of these lncRNAs.

When a lncRNA competes with an mRNA for binding to the same miRNA, the lncRNA and the mRNA both are the targets of the miRNA. We examined the seed regions of hsa-miR-451a to see whether its target mRNAs or lncRNAs are complementary to the seed region in sequence [21]. ENSG00000272620 is perfectly complementary to the seed region of hsa-miR-451a, and mRNA *DLX6* is complementary to the seed region of the hsa-miR-451a with one mismatch pair. This suggests that lncRNA ENSG00000272620 and mRNA *DLX6* should be very likely the targets of hsa-miR-451a. Fig. *S*3 (in the Additional file 1) shows the binding region of lncRNA ENSG00000272620 and hsa-miR-451a and the binding region of mRNA *DLX6* and hsa-miR-451a.

Table 1 shows the top 5 competition scores of the crosstalks mediated by hsa-miR-451a and hsa-miR-375, as calculated by our pointwise mutual information method. A different ceRNA network has a different competition score. Some of the ceRNA competition scores may be similar. For example, the largest competition score of the ceRNA crosstalk mediated by hsa-miR-451a is equal with the competition score of the ceRNA crosstalk mediated by hsa-miR-375. But some competition score of the ceRNA crosstalk is not very similar. Such as the largest competition score of the ceRNA crosstalk mediated by hsa-miR-21-5p is 0.53 which is larger than the largest competition score of ceRNA crosstalk mediated by hsa-miR-451a. However, if two ceRNA crosstalks are mediated by the same miRNA, the higher competition score of the ceRNA crosstalk is, the more reliable the crosstalk is.

**Table 1:** Top-5 competition scores in the ceRNA crosstalks mediated by hsa-miR-375 and hsa-miR-451a

lncRNA	miRNA	mRNA	Score	*P*-value
ENSG00000277199	hsa-miR-375	*GFRAL*	0.35	6.76∗10^−236^
ENSG00000238099	hsa-miR-375	*C6orf58*	0.35	8.48∗10^−228^
ENSG00000279204	hsa-miR-375	*SOX17*	0.31	1.51∗10^−184^
ENSG00000229108	hsa-miR-375	*DUXA*	0.30	2.56∗10^−171^
ENSG00000277199	hsa-miR-375	*MEOX2*	0.30	3.27∗10^−167^
ENSG00000272620	hsa-miR-451a	*DLX6*	0.35	8.88∗10^−45^
ENSG00000279184	hsa-miR-451a	*ZG16*	0.32	1.60∗10^−37^
ENSG00000272620	hsa-miR-451a	*INSM1*	0.31	3.89∗10^−35^
ENSG00000272620	hsa-miR-451a	*NTSR1*	0.30	4.92∗10^−33^
ENSG00000272620	hsa-miR-451a	*GPR26*	0.30	4.92∗10^−33^

### ceRNA networks and breast cancer treatment

The ceRNA crosstalks mediated by hsa-miR-375 or by hsa-miR-451a may regulate the development of breast cancer. These ceRNA crosstalks should be considered in the future for the treatment plan of breast cancer.

As suggested in the third row of Table 1, ENSG00000279204 competes with *SOX17* for binding to hsa-miR-375. *SOX17* is a member of the SRY-related HMG-box family that can regulate cell development [22]. Fu. *et al* found that increasing the expression level of this gene can slow down the speed of breast cancer growth; but reducing the expression level of this gene can lead to poor survival outcomes in breast cancer patients [23]. Thus *SOX17* can be a useful biomarker for breast cancer patients. It can be also understood that the expression of *SOX17* can be up-regulated with the increase of the expression of ENSG00000279204. A high expression level of *SOX17* would lead to decreased growth of breast cancer cell so as to improve the treatment of breast cancer patients.

The gene *MEOX2* is also called *GAX* or *MOX2*. This gene is down-regulated in breast cancer [24]. Recent research shows that *MEOX2* can up-regulate *p21* which is very important for breast tumor grading [25]. Highly expressed *p21* prevents the growth of breast cancer [26]. As shown in the fifth line of Table 1, ENSG00000229108 competes with *MEOX2* for binding with hsa-miR-375. The high expression level of *MEOX2* can enhance the growth of breast cancer. Therefore, decreasing the expression level of ENSG00000229108 can reduce the expression level of *MEOX2*. Thus the high expression level of *MEOX2* would inhibit the growth of breast cancer.

In the last second line of Table 1, ENSG00000272620 competes with *NTSR1* for binding with hsa-miR-451a. *NTSR1* is a target of the Wnt/APC oncogenic pathways which is involved in cell proliferation and transformation [27]. Dupouy found that highly expressed *NTSR1* is associated with the size, the number of metastatic lymph nodes, and Scarff-Bloom-Richardson grading [28]. These suggest that *NTSR1* is a promising target for breast cancer treatment. According to the predicted results, decreasing the expression level of ENSG00000272620 can decrease the expression level of *NTSR1*. Low expression level of *NTSR1* is beneficial for the treatment of breast cancer.

Most breast cancer patients die because of the “incurable” nature of the metastasis breast cancer [29]. About 90% of breast cancer deaths are due to metastasis; indeed, only 20% of the metastatic breast cancer patients can survive more than 1 year [30]. Therefore, inhibiting breast cancer metastasis is very crucial for breast cancer treatment. Morini found that *DLX6* involves in the metastasis potential of breast cancer [31]. Prest also pointed out that *TFF1* can promote breast cancer cell migration [32]. These studies imply that *DLX6* and *TFF1* are highly related to breast cancer metastases. Therefore, decreasing the expression level of these two genes can inhibit breast cancer metastasis. According to our results, lncRNA ENSG00000272620 and ENSG00000279184 cross-regulate *DLX6* and *TFF1* via hsa-miR-451a, respectively. Decreasing the expression level of ENSG00000272620 and ENSG00000279184 can decline the expression levels of *DLX6* and *TFF1*. The low expression levels of these two genes would prevent the development of metastatic breast cancer.

### Roles of ceRNA networks in KEGG pathways

Some lncRNAs can cross-regulate genes which are involved in Kyoto Encyclopedia of Genes and Genomes (KEGG) pathways. Enrichr [33], a gene enrichment analysis web server, is applied to find out these KEGG pathways [34]. 14 KEGG pathways are found with *p*-values lower than 0.05. Some of these KEGG pathways are the key pathway in regulating breast cancer and may be a potential drug target for breast cancer treatment, such as the chemokine signaling pathway, the cytokine-cytokine receptor interaction, and the neuroactive ligand-receptor interaction [35*–*37]. All the KEGG pathways are presented in Table. *S*4 (in the Additional file 1). In this subsection, we focus on analyzing the chemokine signaling pathway.

**Fig. 2:**
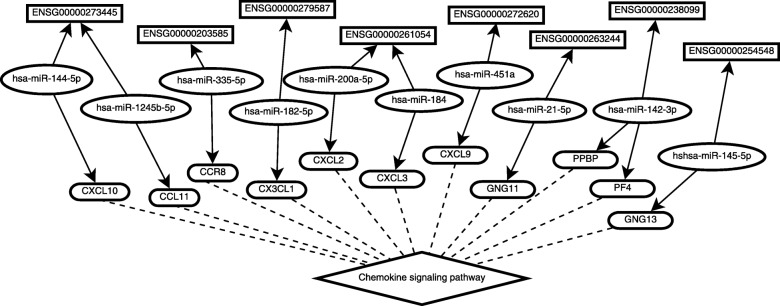
The ceRNA networks involved in the chemokine signaling pathway

The cross regulation between the lncRNAs and the genes involved in the chemokine signaling pathway is shown in Fig. 2, demonstrating 11 genes related to chemokine signaling pathway are involved in breast cancer. Of them, *CXCL10*, *CXCL9*, *CCL11*, *CCR8*, and *GNG13* up-regulate breast cancer, while the other genes download-regulate breast cancer. Chemokine signaling pathway expresses on the immune cells and regulates immune responder. However, new evidences show that the gene in the chemokine signaling pathway also plays a vital role in breast cancer progression [36]. For example, *CXCL10* affects the tumor microenvironment and plays important role in breast cancer progression [38], *CXCL9* is identified as a biomarker in breast cancer [39]. Regulating these gene can inhibit the growth of breast cancer.

### A ceRNA which may be an efficient drug target for breast cancer treatment

**Fig. 3:**
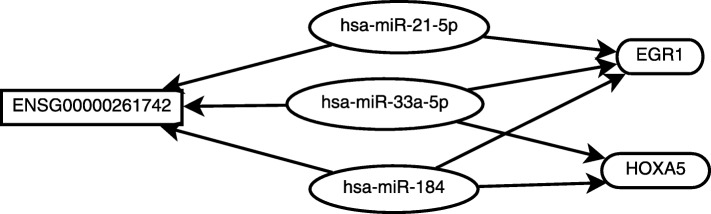
A ceRNA network cross-regulates two mRNAs through three miRNAs

Two different miRNAs may have common target mRNAs and common target lncRNAs. A common target lncRNA can cross-regulate mRNAs through different miRNAs. Therefore, this common target lncRNA is an efficient drug target for cancer treatment. An example can be found in Fig. 3. The lncRNA ENSG00000261742 competes for binding to hsa-miR-21-5p, hsa-miR-33a-5p, and hsa-miR-184 with *HOXA5* and *EGR1*. *EGR1* is known to up-regulate *PTEN* which is a key tumor breast suppressor gene [40]. It implies that increasing the expression level of *EGR1* can suppress the development of breast cancer. The lowly expressed *HOXA5* lead to the functional activation of twist and promoting the development of breast cancer [41]. Therefore, increasing the expression level of these two mRNAs are very important for breast cancer treatment.

Hsa-miR-21-5p, hsa-miR-33a-5p, and hsa-miR-184 can regulate the expression of these two mRNAs. However, only decreasing the expression level of one miRNA cannot enhance the expression levels of these two mRNAs, since the high expression of the other miRNA can decrease the expression of both mRNAs. In our results, increasing the expression of ENSG00000261742 can enhance the expression of these two mRNAs by decreasing the expression of these two miRNAs. Therefore, ENSG00000261742 is an efficient drug target for increasing the expression of both mRNAs. About all, this ceRNA is suggested to be an efficient drug target for breast cancer treatment.

## Discussion

The ceRNA hypothesis is still in its infancy, many ceRNA networks have not been discovered yet. The mutations of miRNA may change existing or lead to new crosstalk. For example, the 5^′^ variant of miRNA may bind to different target mRNA or lncRNA comparing to its wildtype miRNA since the shift of the seed region of the miRNA. Further, the ceRNA hypothesis illustrates the complexity of RNA regulatory network. By this hypothesis, some other complexity networks may exist. Our method for discovering ceRNA network from the RNA-seq data that contains the expression level of RNA (miRNA, lncRNA, and mRNA) is limited to only the tumor and normal tissues, how to incorporate different tissues that have a matching RNA and miRNA sequencing data set to extend our analysis is a future direction of our research in this area.

A lncRNA that is not differentially expressed may contribute to the sponge mechanism as well [42]. In particular, the relative concentration of the ceRNAs and changes in the ceRNA expression levels are very important for discovering ceRNA networks [5]. Indeed, conditions like the relative concentration of ceRNAs and their microRNAs or other conditions not necessarily corresponding to differentially expressed RNAs can be applicable as starting points to discover ceRNAs. These will be some of our future work to enrich the ceRNA sponge hypothesis.

## Conclusion

In this paper, we proposed a novel method for constructing ceRNA networks from paired RNA-seq data sets. We first identify the differentially expressed lncRNAs, miRNAs, and mRNAs from the paired RNA-seq data sets. Then we derive the competition regulation mechanism from the competition rule and construct the candidate ceRNA crosstalks based on this rule. This competition regulation mechanism is another feature of the ceRNA network and is useful for constructing ceRNA networks. Finally, the pointwise mutual information is applied to measure the competitive relationship between these RNAs to select reliable ceRNA crosstalks to construct the ceRNA networks. The analysis results have shown that the function of ceRNA networks is related to the growth, proliferation, and metastatic of breast cancer. These ceRNA networks present the complex regulatory mechanism of the RNAs in breast cancer. In addition, the ceRNA networks suggest a new approach for breast cancer treatment.

## Method

**Fig. 4:**
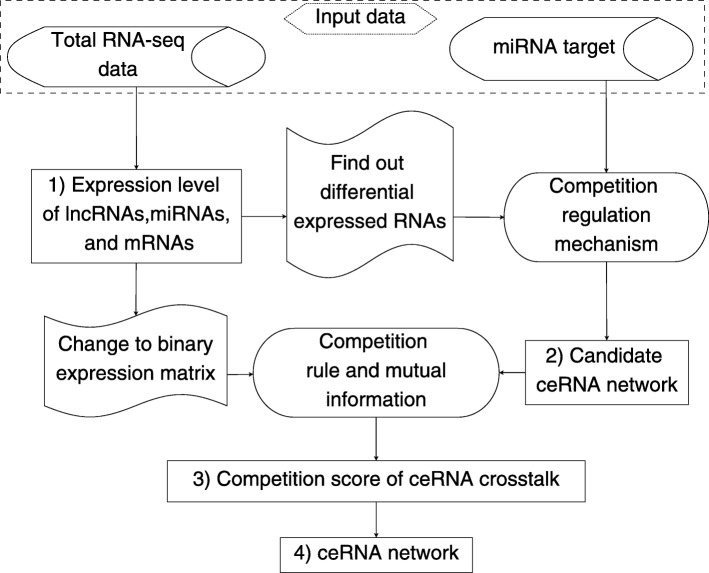
The framework of our method

Our method for constructing ceRNA network has four steps. Firstly, it computes the expression levels of lncRNA, miRNA, and mRNA from the breast cancer tumor tissues and normal tissues. Secondly, the predicted miRNA targets, differentially expressed RNAs, and the competition regulation mechanism are used to construct the candidate ceRNA networks. Thirdly, it combines the competition rule and the pointwise mutual information to compute the competition score of each ceRNA crosstalk. Finally, we select the ceRNA crosstalks which have significant competition scores to construct the ceRNA network. Fig. 4 shows the framework of our method.

### Definitions and data preprocessing

**Fig. 5:**
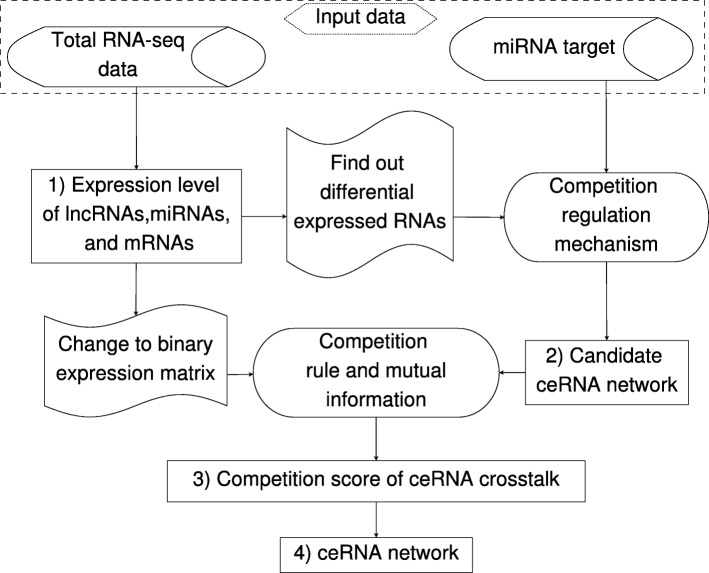
**a** A ceRNA crosstalk; **b** A ceRNA network

If a lncRNA *lnc* competes with an mRNA *mr* for binding to an miRNA *mir*, the triple of *lnc*, *mir*, and *mr* is called a ceRNA crosstalk denoted by *T*=(*lnc*,*mir*,*mr*). We also say that ceRNA crosstalk *T*=(*lnc*, *mir*, *mr*) is mediated by *mir*. For example, Fig. 5a is a ceRNA crosstalk *T*=(*lncRNA*_1_, *miRNA*, *mRNA*_1_) mediated by *miRNA*.

All the ceRNA crosstalks mediated by the same miRNA as a whole is defined as a ceRNA network. It is denoted by *N*=(*lnR*, *mir*, *mR*), where *lnR* stands for the set of lncRNAs, *mir* is the miRNA, and the *mR* stands for the set of mRNAs. We also say ceRNA network *N*=(*lnR*,*mir*,*mR*) is mediated by *mir*. For example, Fig. 5b is a ceRNA network, where *lnR*={*lncRNA*_1_,*lncRNA*_2_,…,*lncRNA*_*n*_} and *mR*={*mRNA*_1_,*mRNA*_2_,…,*mRNA*_*m*_}.

The paired breast cancer RNA-seq data set was downloaded from the TCGA GDC data portal website [43]. This paired data set contains the expression levels of lncRNAs, mRNAs, and miRNAs of 102 tumor and normal tissue samples. The TCGA IDs of these 102 samples are listed in Additional file 1: Table S5. These RNAs and their expression levels form an expression matrix. Table *S*1 is an example of expression matrix. Some RNAs expresses in only a few tissue samples. These low frequently expressed RNAs are not important for breast cancer study and may have noise affect to the result. Thus, these RNAs which are not expressed in half of the whole tissue samples were removed from the expression matrix. We transform the expression matrix to a binary expression matrix by using the equal frequency discretization method: for the same RNA expressed in all samples, if this RNA expression level of a sample is higher (lower) than the median RNA expression level of all the samples, this RNA is highly (lowly) expressed in this sample and is assigned with binary value 1 (0). This process was conducted using Weka3.8 [44].

Let *I*[*R*,*S*] denotes the binary expression matrix, where *R* is the set of RNAs from the original data set after the noise removal, and *S* is the set of samples. In the binary expression matrix, 1 represents that the expression level of the RNA is relatively high, 0 means that the expression level of the RNA is relatively low. Table *S*2 is the binary expression matrix transformed from Table *S*1.

For a given binary expression matrix *I*[*R*,*S*], we define that *r*^′^ is a RNA from *R* and *sa*^′^ is a sample from *S*. *I*[*r*^′^,*sa*^′^] is the value of the RNA *r*^′^ of the sample *sa*^′^ in the binary expression matrix *I*[*R*,*S*]. For example, in Table *S*2, *I*[*lnc*_1_,*sa*_1_] is 0 and *I*[*mr*_*m*_,*sa*_2_] is 1.

### Constructing a candidate ceRNA network

The target mRNAs and lncRNAs of the miRNAs were downloaded from the miRWalk2.0 database [45]. The miRWalk2.0 database contains the comparison results of binding sites from 12 existing miRNA-target prediction software tools [46]. It is a high quality database of miRNA targets. Also, this database contains the miRNA’s target lncRNAs and target mRNAs. An miRNA (with *p*-value ≤0.05 and absolute fold change ≥2.0), its target lncRNAs (with *p*-value ≤0.05 and absolute fold change ≥3.0) and its target mRNAs (with *p*-value ≤0.05 and absolute fold change ≥2.0) are used to construct the initial ceRNA network. The differentially expressed lncRNA, miRNA, and mRNA are computed by using fold change [47] and the t-test method [48].

Suppose a lncRNA *lnc*, an miRNA *mir*, and an mRNA *mr* form a ceRNA crosstalk. If *lnc* up-regulates in breast cancer samples, then the fold change of *lnc* should be larger than 0. According to the competition rule, the highly expressed lncRNA can lead to low expression of the miRNA, i.e., *mir* down-regulates and the fold change of *mir* should be smaller than 0. The low expression level of the miRNA increases the expression level of the mRNA. Therefore, *mr* up-regulates in the breast cancer samples, and the fold change of *mr* should be larger than 0. Similarly, if *lnc* down-regulates and the fold change of *lnc* is smaller than 0, then *mir* up-regulates in the breast cancer samples and the fold change of *mir* should be larger than 0. Then *mr* down-regulates in the breast cancer tumor and the fold change of *mr* is smaller than 0. Based on this principle, we propose a competition regulation mechanism. This competition regulation mechanism is divided into a positive and a negative competition regulation facet:

- Positive competition regulation mechanism: the fold change of the miRNA is larger than 0, and the fold changes of lncRNAs and mRNAs are smaller than 0.
- Negative competition regulation mechanism: the fold change of the miRNA is smaller than 0, the fold changes of lncRNAs and mRNAs are larger than 0.

Given the initial ceRNA network, we find the lncRNAs and mRNAs which follow the positive or negative competition regulation mechanism. Then the miRNA, the rest of the lncRNAs and mRNAs construct a candidate ceRNA network. We denote the candidate ceRNA network by *N*^′^=(*lncR*, *mir*, *mR*), where *lncR* and *mR* stand for the sets of lncRNAs or mRNAs which follow the competition regulation mechanism.

### Computing the competition score

A candidate ceRNA network is formed by combining many ceRNA crosstalks. Some of these candidate ceRNA crosstalks may not satisfy the competitive relationship. Pointwise mutual information was proposed to measure the relationships between individual words in a corpus [49]. If two words frequently co-occur, the pointwise mutual information is high. In this work, we apply it to measure the competitive relationships between RNAs in a ceRNA network, namely if a lncRNA can cross regulate an mRNA through an miRNA, the pointwise mutual information of this crosstalk should be high. Traditional pointwise mutual information utilizes the probability coincidence or Gaussian kernel to measure the relationship between the variables; and only a positive or only a negative score between the variables is calculated. However, the competitions in a ceRNA crosstalk have both negative and positive relationships between the two RNAs. Therefore, the traditional pointwise mutual information needs to be refined for measuring the competition relationships between the RNAs in a ceRNA crosstalk. In this work, we calculate the pointwise mutual information based on our competition rule, as detailed below.

Given a candidate ceRNA network *N*^′^=(*lncR*, *mir*, *mR*), where *lncR*={*lnc*_1_, *lnc*_2_, …, *lnc*_*n*_} and *mR*={*mr*_1_, *mr*_2_, …, *mr*_*m*_}, any lncRNA *lnc*_*i*_∈*lncR*, *mir*, and any mRNA *mr*_*j*_∈*mR* can form a ceRNA crosstalk *T*=(*lnc*_*i*_, *mir*, *mr*_*j*_). We use a competition score to measure the reliability of each ceRNA crosstalk. The higher the competition score of the ceRNA crosstalk is, the more reliable the ceRNA crosstalk is.

Given a binary expression matrix *I*[*R*,*S*], let *lnc*_*i*_, *mir*, and *mr*_*j*_ be a lncRNA, an miRNA, and an mRNA of *R*, respectively, and let *sa*_*l*_ be one of the samples in *S*. If *lnc*_*i*_, *mir*, and *mr*_*j*_ in *sa*_*l*_ are satisfied with one of these conditions:

- Condition 1: *I*[*lnc*_*i*_,*sa*_*l*_]=0, *I*[*mir*,*sa*_*l*_]=1, and *I*[*mr*_*j*_,*sa*_*l*_]=0.
- Condition 2: *I*[*lnc*_*i*_,*sa*_*l*_]=1, *I*[*mir*,*sa*_*l*_]=0, and *I*[*mr*_*j*_,*sa*_*l*_]=1.

we say that *sa*_*l*_ is the competition sample of *T*=(*lnc*_*i*_,*mir*,*mr*_*j*_). For example, at Table *S*2, *sa*_1_ is a competition sample of *T*=(*lnc*_1_,*mir*_1_,*mr*_1_), since *I*[*lnc*_1_,*sa*_1_]=0, *I*[*mir*_1_,*sa*_1_]=1, and *I*[*mr*_1_,*sa*_1_]=0. In addition, we define that *supp*^*S*^(*lnc*_*i*_,*mir*,*mr*_*j*_) is the total number of the competition samples of *T*=(*lnc*_*i*_,*mir*,*mr*_*j*_) in the sample set *S*.

The competition score of *T*=(*lnc*_*i*_,*mir*,*mr*_*j*_) is computed by using pointwise mutual information:

$$ \begin{aligned} {PMI}_{mir}^{S}({lnc}_{i},{mr}_{j}) = log \frac{P_{mir}^{S}({lnc}_{i},{mr}_{j})}{P_{mir}^{S}({lnc}_{i})P_{mir}^{S}({mr}_{j})} \end{aligned} $$

where $P_{mir}^{S}({lnc}_{i},{mr}_{j})$, $P_{mir}^{S}({lnc}_{i})$, and $P_{mir}^{S}({mr}_{j})$ are computed by:

$$ \begin{aligned} &P_{mir}^{S}({lnc}_{i},{mr}_{j}) = \frac {supp^{S}({lnc}_{i},mir,{mr}_{j})} {\sum_{i^{\prime}=1}^{n}\sum_{j^{\prime}=1}^{m}supp^{S}({lnc}_{i^{\prime}},mir,{mr}_{j^{\prime}})} \\ &P_{mir}^{S}({lnc}_{i}) = \frac {\sum_{j^{\prime}=1}^{m}supp^{S}({lnc}_{i},mir,{mr}_{j^{\prime}})} {\sum_{i^{\prime}=1}^{n}\sum_{j^{\prime}=1}^{m}supp^{S}({lnc}_{i^{\prime}},mir,{mr}_{j^{\prime}})} \\ &P_{mir}^{S}({mr}_{j}) = \frac {\sum_{i^{\prime}=1}^{n}supp^{S}({lnc}_{i^{\prime}},mir,{mr}_{j})} {\sum_{i^{\prime}=1}^{n}\sum_{j^{\prime}=1}^{m}supp^{S}({lnc}_{i^{\prime}},mir,{mr}_{j^{\prime}})} \\ \end{aligned}  $$

A positive pointwise mutual information means the variables co-occur more frequently than what would be expected under an independence assumption, and a negative pointwise mutual information means the variables co-occur less frequently than what would be expected.

### Selecting a crosstalk which has a significant competition score

A competition score can be 0, negative, or positive. If the competition score of a ceRNA crosstalk is 0 or negative, it implies that there is no competitive relationship between the lncRNA, miRNA, and mRNA or the competitive relationship is less reliable than we would be expected. Such a ceRNA crosstalk should be discarded. A positive competition score indicates that the competitive relationship between these RNAs is more reliable than what we expected, and thus the ceRNA crosstalk is reliable to construct the ceRNA network. Further, the higher the competition score, the more reliable the ceRNA crosstalk is. Therefore, we should select those crosstalks which are reliable enough to construct the ceRNA network.

Suppose we are given *t* candidate ceRNA crosstalks and their competition scores are {*PMI*_1_, *PMI*_2_, …, *PMI*_*t*_} which are all positive. A threshold *θ* is applied to distinguish low and high competition scores, and the problem is to reject the null hypothesis. The null hypothesis is that the competition score is small, that is, it implies there is no competing relationship in this crosstalk. If the competing score is very high, the null hypothesis can be rejected—it implies that this ceRNA crosstalk involves in regulating the biological process. For a ceRNA crosstalk *a*, its significance level *θ*_*a*_ of the competition score is:

$$\theta_{a} = \frac{PMI_{a}-\overline{PMI}}{\sigma} $$

where $\overline {PMI}$ and *σ* are the average and standard deviation of the entire competition scores. The *p*-value of the ceRNA crosstalk *a* is $p_{a}=erfc(\theta _{a}/\sqrt {2})$ [50]. If the *p*-value of a ceRNA crosstalk is lower than 0.05, this ceRNA crosstalk has significant competition score. We select those ceRNA crosstalks which have significant competition scores to construct the ceRNA network.

The novelty of our method is to apply competition regulation mechanism to construct candidate ceRNA networks and utilize the pointwise mutual information to calculate the competition scores. The competition regulation mechanism, which is deducted from the competition rule, reflects the nature of the competition rule. Therefore, this regulation mechanism is a critical feature of the ceRNA network and can be applied to filter out many noisy eRNAs. Pointwise mutual information can measure both non-linear and linear relationship, and it is suitable for calculating the competition score of ceRNA crosstalks. Further, our method utilizes the pointwise mutual information to measure the point-to-point competitive relationships between lncRNA, miRNA, and mRNA, but not the pair-wise relationship between the two RNAs.

## Supplementary information

**Additional file 1** Comprehensive Comparison with Other Methods, Supplementary Figures and Tables.

## Data Availability

The results and the Python source code of our algorithm can be downloaded from the website https://github.com/ChaowangLan/ceRNA.

## Abbreviations

ceRNA: Competing endogenous RNA
KEGG: Kyoto encyclopedia of genes and genomes
lncRNA: Long noncoding RNA
