# Supplementary material for: Construction of competing endogenous RNA networks from paired RNA-seq data sets by pointwise mutual information
Source: BMC Genomics. 2019 Dec 24;20(Suppl 9):943. doi: 10.1186/s12864-019-6321-x (PMC6929403; doi:10.1186/s12864-019-6321-x)
Supplement: Supplementary file 1 — Additional file 1 Comprehensive Comparison with Other Methods, Supplementary Figures and Tables. [file 12864_2019_6321_MOESM1_ESM.pdf]

Construction of Competing Endogenous RNA Networks from Paired RNA-seq Data Sets by Pointwise Mutual Information (Supplementary Material)

Chaowang Lan[1], Hui Peng[1], Gyorgy Hutvagner[2], and Jinyan Li*[1]

[1]Advanced Analytics Institute, Faculty of Engineering and IT, University of Technology Sydney, NSW 2007, Australia

[2]Centre for Health Technologies, University of Technology Sydney, NSW 2007, Australia

November 12, 2019

Comprehensive Comparison with Other Methods

We compared our prediction results with three existing methods. The first comparison is with Chen's method [1] which is based on the idea of Pearson correlation coefficient. The second comparison is with Paci's method [3] which is a partial correlation method. The third comparison is with Sumazin's method [5] which is based on the conditional mutual information. Our method, Chen's method, Paci's method, and Sumazin's method predicted total 30365, 106045, 15420, and 227755 ceRNA crosstalks, respectively. Sumanzin's method identified the most. Figure S1 is a Venn graph showing the common and unique ceRNA crosstalks predicted by these methods. Each boundary line encloses a number of ceRNA crosstalks predicted by one or more methods; and the intersection areas indicate the numbers of common ceRNA crosstalks.

Note that 26620 ceRNA crosstalks predicted by our method are also identified by one of the three existing methods. Our method have more common ceRNA crosstalks in comparison with Sumazin's method (21095 of our predicted ceRNA crosstalks) than the other methods (1168 and 16153 of our predicted ceRNA crosstalks are identified by the Paci's method and Chen's method, respectively). However, 3745 ceRNA crosstakls predicted by our method are not identified by the other methods.

Some of these ceRNA crosstalks may regulate breast cancer processes. For example, the ceRNA crosstalk ($ENSG00000272620$, $hsa\text{-}miR\text{-}451a$, $GPR26$), which had been showed in Table 1, was able to be predicted by our method only. Gene $GPR26$ is a member of G-protein-coupled receptors. The G-protein-coupled receptors can play key roles in tumorigenesis, angiogenesis, and metastasis [4]. The ceRNA crosstalk ($ENSG00000250266$, $hsa\text{-}miR\text{-}142\text{-}3p$, $PF4$) is also predicted by our method but not identified by the other methods. The highly expressed lncRNA ENSG00000272620 may compete and cross regulate $GPR26$ for binding to hsa-miR-451a to influence breast cancer tumorigenesis, angiogenesis, and metastasis. The lowly expressed lncRNA ENSG00000250266 could not down-regulate the hsa-miR-142-3p and might lead to lowly expressed $PF4$. Lowly expressed $PF4$ could not suppress breast cancer growth [2]. LncRNA ENSG00000250266 may be a potential target for breast cancer treatment.

Many methods, including Paci's method, identify ceRNA networks only taking into account the expression data. These methods could find all the negative and positive expression relationships between the RNAs. It seems that these methods are unbiased and preferable to identify ceRNA networks. However, the competition relationship between RNAs is a specific relationship (i.e., lncRNA and miRNA are negatively co-expressed; miRNA and mRNA are negatively co-expressed). Thus, ceRNA networks should all hold this specific relationship.

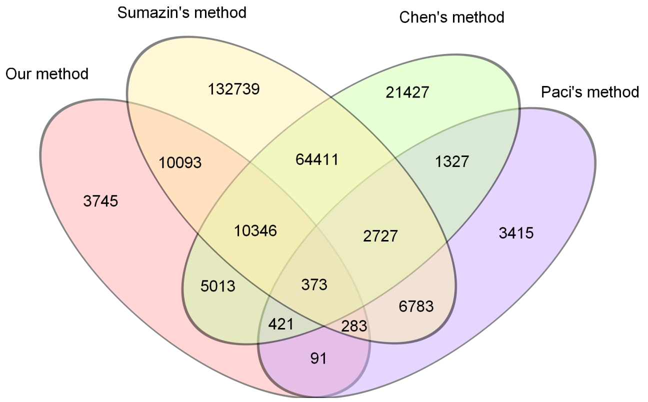

Figure S1: The common and unique ceRNA crosstalks predicted by various methods

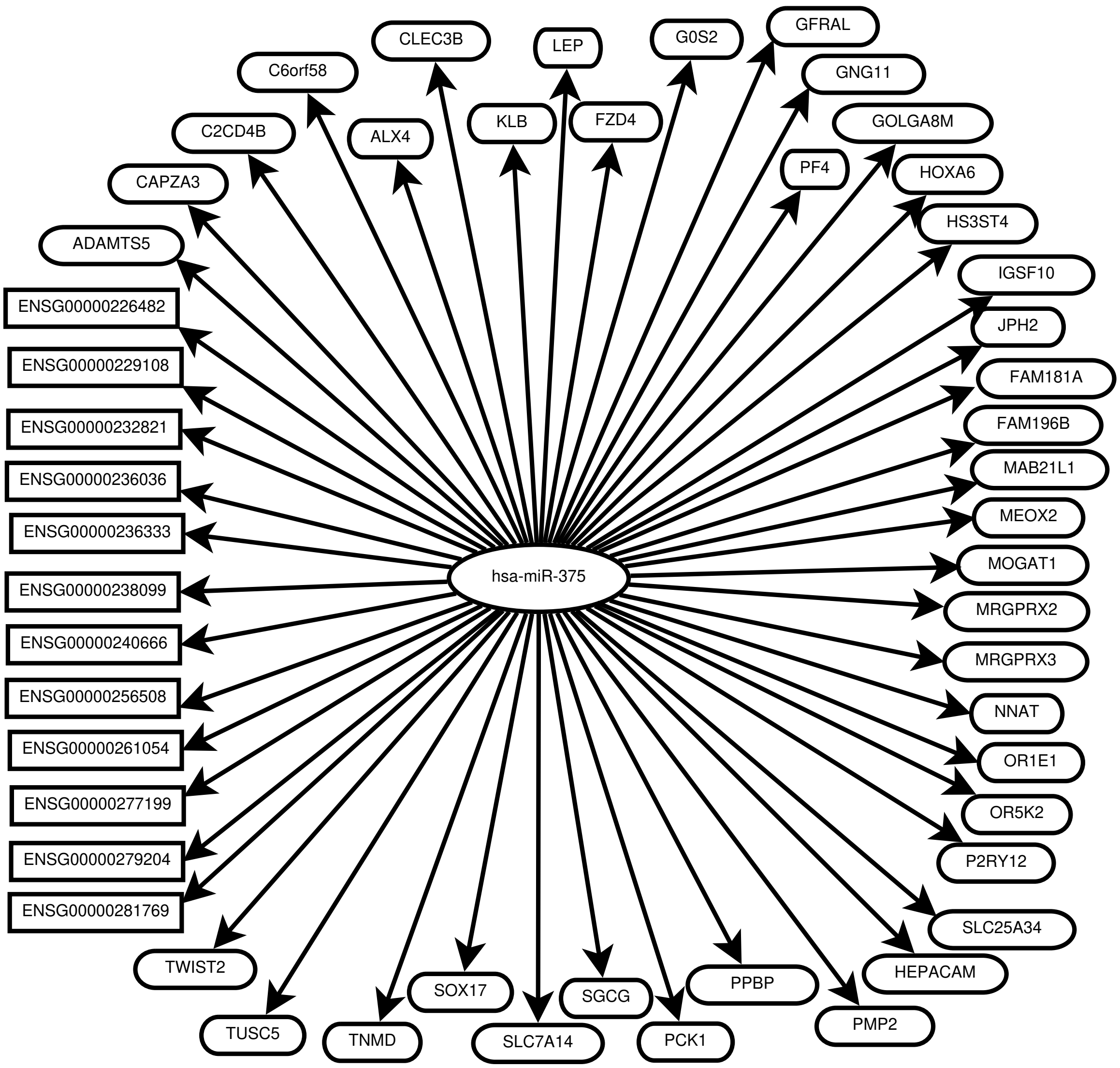

Figure S2: The ceRNA network formed from the top 50 candidate ceRNA crosstalks mediated by hsa-miR-375. Text words in the rectangle boxes are the names of the lncRNAs and text words in the oval boxes are the names of the mRNAs.

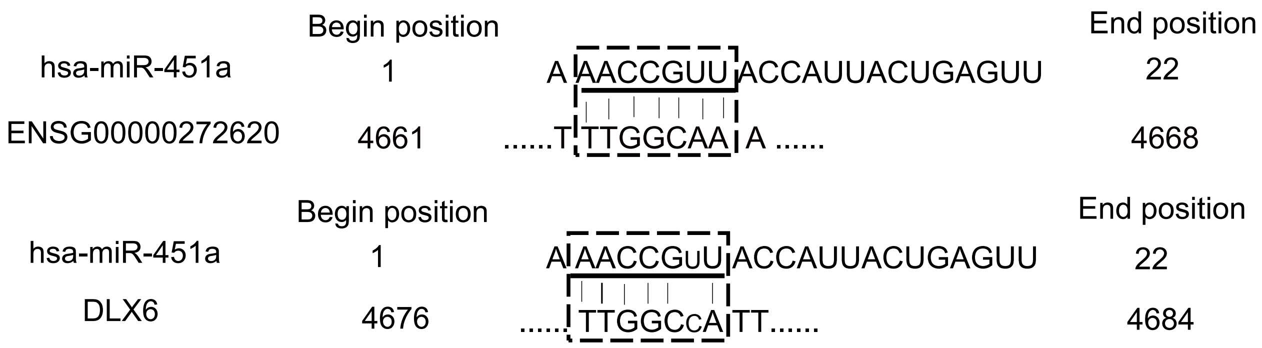

Figure S3: The binding sites of lncRNA, miRNA, and mRNA

Table S1: A matrix of expression levels of RNAs

	sa_1	sa_2	...	sa_s
lnc_1	40	50	...	70
...	...	...	...	...
lnc_n	10	15	...	33
mir_1	450	350	...	150
...	...	...	...	...
mir_k	500	700	...	600
mr_1	20	30	...	50
...	...	...	...	...
mr_m	65	85	...	25

Table S2: The binary expression matrix of RNAs transformed from Table S1

	sa_1	sa_2	...	sa_s
lnc_1	0	0	...	1
...	...	...	...	...
lnc_n	0	0	...	1
mir_1	1	1	...	0
...	...	...	...	...
mir_k	0	1	...	1
mr_1	0	0	...	1
...	...	...	...	...
mr_m	1	1	...	0

Table S3: Expression fold change ratios and p-values of the lncRNAs involved in the ceRNA networks mediated by hsa-miR-451a and hsa-miR-375

LncRNA	Fold change	p-value
ENSG00000226482	-4.19	$7.46 * 10^{-9}$
ENSG00000227260	-3.21	$3.18 * 10^{-26}$
ENSG00000229108	-3.76	$8.50 * 10^{-20}$
ENSG00000232821	-3.03	$2.33 * 10^{-12}$
ENSG00000236036	-3.25	$6.19 * 10^{-8}$
ENSG00000236333	-4.88	$2.11 * 10^{-22}$
ENSG00000238099	-3.20	$4.66 * 10^{-16}$
ENSG00000240666	-3.35	$1.41 * 10^{-18}$
ENSG00000256508	-3.55	$9.48 * 10^{-29}$
ENSG00000261054	-3.14	$2.17 * 10^{-18}$
ENSG00000277199	-3.48	$7.06 * 10^{-7}$
ENSG00000279204	-3.35	$4.92 * 10^{-18}$
ENSG00000281769	-4.10	$4.24 * 10^{-6}$
ENSG00000263655	4.62	$2.27 * 10^{-7}$
ENSG00000272620	3.86	$7.36 * 10^{-3}$
ENSG00000279184	3.31	$2.04 * 10^{-5}$

Table S4: KEGG pathways which can be regulated by ceRNA networks

KEGG name	P-value	Number of gene
Alcoholism	$3.62 * 10^{-19}$	28
Systemic lupus erythematosus	$4.48 * 10^{-19}$	25
Viral carcinogenesis	$5.04 * 10^{-5}$	13
Cytokine-cytokine receptor interaction	$1.84 * 10^{-4}$	14
Chemokine signaling pathway	$3.62 * 10^{-4}$	11
Transcriptional misregulation in cancer	$3.69 * 10^{-3}$	9
Salivary secretion	$4.06 * 10^{-3}$	6
Neuroactive ligand-receptor interaction	$7.92 * 10^{-3}$	11
Serotonergic synapse	$1.21 * 10^{-2}$	6
Oxytocin signaling pathway	$1.84 * 10^{-2}$	7
Morphine addiction	$1.93 * 10^{-2}$	5
Circadian entrainment	$2.28 * 10^{-2}$	5
Renin secretion	$2.32 * 10^{-2}$	4
Retrograde endocannabinoid signaling	$2.88 * 10^{-2}$	5

References

[1] J. Chen, J. Xu, Y. Li, J. Zhang, H. Chen, J. Lu, Z. Wang, X. Zhao, K. Xu, Y. Li, et al. Competing endogenous RNA network analysis identifies critical genes among the different breast cancer subtypes. *Oncotarget*, 8(6):10171, 2017.

[2] S. N. M. Nafi, F. Idris, and H. Jaafar. Cellular and Molecular Changes in MNU-Induced Breast Tumours Injected with PF4 or bFGF. *Asian Pacific Journal of Cancer Prevention: APJCP*, 18(12):3231, 2017.

[3] P. Paci, T. Colombo, and L. Farina. Computational analysis identifies a sponge interaction network between long non-coding RNAs and messenger RNAs in human breast cancer. *BMC Systems Biology*, 8(1):83, 2014.

[4] A. Singh, J. J. Nunes, and B. Ateeq. Role and therapeutic potential of G-protein coupled receptors in breast cancer progression and metastases. *European Journal of Pharmacology*, 763:178–183, 2015.

[5] P. Sumazin, X. Yang, H.-S. Chiu, W.-J. Chung, A. Iyer, D. Llobet-Navas, P. Rajbhandari, M. Bansal, P. Guarnieri, J. Silva, et al. An extensive microRNA-mediated network of RNA-RNA interactions regulates established oncogenic pathways in glioblastoma. *Cell*, 147(2):370–381, 2011.
